# Early economic evaluation of emerging health technologies: protocol of a systematic review

**DOI:** 10.1186/2046-4053-3-81

**Published:** 2014-07-23

**Authors:** Ba’ Pham, Hong Anh Thi Tu, Dolly Han, Petros Pechlivanoglou, Fiona Miller, Valeria Rac, Warren Chin, Andrea C Tricco, Mike Paulden, Joanna Bielecki, Murray Krahn

**Affiliations:** 1 Toronto Health Economics and Technology Assessment Collaborative, University of Toronto, Leslie Dan Pharmacy Building, 6th floor, Room 651, 144 College Street, Toronto, Ontario M5S 3M2, Canada; 2 Institute of Health Policy, Management and Evaluation, University of Toronto, 155 College St, Toronto, Ontario M5S 3M2, Canada; 3 ILEX Consulting Inc, Toronto, Ontario M4W 3Y6, Canada; 4 Li Ka Shing Knowledge Institute of St. Michael's Hospital, Toronto, Ontario M5B 1W8, Canada; 5 Department of Emergency Medicine, University of Alberta, Edmonton, Alberta T6G 2R3, Canada; 6 Faculty of Pharmacy, University of Toronto, Toronto, Ontario M5S 3M2, Canada

## Abstract

**Background:**

The concept of early health technology assessment, discussed well over a decade, has now been collaboratively implemented by industry, government, and academia to select and expedite the development of emerging technologies that may address the needs of patients and health systems. Early economic evaluation is essential to assess the value of emerging technologies, but empirical data to inform the current practice of early evaluation is limited. We propose a systematic review of early economic evaluation studies in order to better understand the current practice.

**Methods/design:**

This protocol describes a systematic review of economic evaluation studies of regulated health technologies in which the evaluation is conducted prior to regulatory approval and when the technology effectiveness is not well established. Included studies must report an economic evaluation, defined as the comparative analysis of alternatives with respect to their associated costs and health consequences, and must evaluate some regulated health technology such as pharmaceuticals, biologics, high-risk medical devices, or biomarkers. We will conduct the literature search on multiple databases, including MEDLINE, EMBASE, the Centre for Reviews and Dissemination Databases, and EconLit. Additional citations will be identified via scanning reference lists and author searching. We suspect that many early economic evaluation studies are unpublished, especially those conducted for internal use only. Additionally, we use a chain-referral sampling approach to identify authors of unpublished studies who work in technology discovery and development, starting out with our contact lists and authors who published relevant studies. Citation screening and full-text review will be conducted by pairs of reviewers. Abstracted data will include those related to the decision context and decision problem of the early evaluation, evaluation methods (e.g., data sources, methods, and assumptions used to identify, measure, and value the likely effectiveness and the costs and consequences of the new technology, handling of uncertainty), and whether the study results adequately address the main study question or objective. Data will be summarized overall and stratified by publication status.

**Discussion:**

This study is timely to inform early economic evaluation practice, given the international trend in early health technology assessment initiatives.

## Background

Economic evaluation is the comparative analysis of alternative technologies with respect to their costs and consequences [1]. It is often used late in the evaluation of drugs, devices, and other technologies to inform coverage decisions [2]. This is problematic because the evidence portfolio at the time of the reimbursement decision is often incomplete. Cost and preference data are often not gathered prospectively within clinical trials designed to evaluate effectiveness. This means that analysts cannot directly use trial data and must struggle to assemble evidence from a variety of sources and settings to evaluate the cost-effectiveness of new interventions [3]. Published data suggest that considerable economic uncertainty existed in about half of the submissions for reimbursement decisions [4]. Also, clinical trial design is rarely informed by *ex ante* economic modeling. This means that optimal strategies and the full range of subgroups may not have been fully considered prior to commencing the clinical research program.

Economic evaluation can be used early in the process of technology evaluation. As an example, Vallejo-Torres et al. illustrate a three-stage cost-effectiveness analysis of a new medical device: an early phase in which simple methods are used to estimate the maximum price attainable for the technology, a mid-stage which synthesizes data into a cost-effective model and identifies which information is most valuable to reduce decision uncertainty, and a late stage in which all relevant information is synthesized [5]. The results suggest that early and iterative economic evaluation could be useful to inform decisions along the technology development process.

Early evaluation can contribute to decision-making by both industry and government [6,7]. From an industry's perspective, early evaluation may be used for early market assessment, managing research and development portfolios, and informing pricing and reimbursement scenarios. From the policy perspective, decision makers may benefit from information supplied by early evaluation. Thus, there is interest from both innovators and payers in early health technology assessment and, more specifically, early economic evaluation, to inform planning and development decisions by industry and to inform the potential of new technologies that may meet health system needs [8].

Though interest in early economic evaluation is considerable, much applied research is still at the pilot stage. There is uncertainty about which technologies can benefit from early evaluation, what methods are appropriate, and the contribution of early economic evaluation to health technology assessment [8]. The aim of this systematic review is to describe the characteristics of early economic evaluation of emerging technologies and to understand current methods to early evaluation.

## Methods/design

This protocol describes a systematic review of economic evaluation studies that are conducted to inform the development and planning of early evaluation of emerging health technologies.

### Inclusion criteria

An economic evaluation is defined as the comparative analysis of alternatives with respect to their associated costs and health consequences. A study is included if it reports an early economic evaluation of a regulated health technology in order to inform the development of the technology (e.g., pre-clinical research or phase I or II studies). For this review, regulated health technologies include pharmaceuticals, biologics, medical devices, or biomarkers. The term ‘biologic’ refers to a class of therapeutics that are produced by means of biological processes, including, for example, the blood production-stimulating protein erythropoietin, a growth-stimulating hormone, a gene therapy, a monoclonal antibody, or a vaccine [9]. The term ‘medical device’ refers to a class II device (e.g., blood pressure monitors, contact lenses, pregnancy test kits, single-use surgical instruments, catheters), a class III device (e.g., ventilators, cardiac monitors, hip implants, knee implants, lasers, chlamydia test kits, glucose meters), or a class IV device (e.g., defibrillators, pacemakers, coronary stents, HIV test kits, neurosurgical shunts) that requires product licensing for general marketing purposes (examples are from a presentation by Ms. Sarah Chandler of Health Canada to MaRS-Excite, September 2012, with permission). The term ‘biomarker’ (or a biological marker) refers to an objectively measured and evaluated indicator of a normal biological process, a pathogenic process (e.g., a DNA sequence that is associated with susceptibility to disease), or a pharmacologic response to a therapeutic intervention [10].

### Literature search

The literature search will be conducted on multiple databases, including MEDLINE (Ovid), MEDLINE In-Process (Ovid), EMBASE (Ovid), the Centre for Reviews and Dissemination: National Health Services Economic Evaluation Database (NHS EED), the Cochrane Library: Health Technology Assessment Database (HTA Database), and EconLit. The first four sources are standard repositories of economic evaluation studies. The last database is a reliable source of citations and abstracts of economic research [11]. All databases will be searched from the date of inception until present.

The search used both Medical Subject Headings (MeSH) as well as keyword variants of all relevant terms, including keyword terms derived from MeSH scope notes to increase retrieval of citations from Medline In-Process. The search developed in MEDLINE will be adopted with the syntax and subject headings appropriate for the other databases mentioned. In addition to early economic evaluation keywords and MeSH terms generated through scoping search (below), the final search strategy will include the validated NHS EED search filter and a published MEDLINE G and EMBASE G filter to identify potentially relevant citations of economic evaluation studies in MEDLINE and EMBASE [12]. Both MEDLINE G and EMBASE G filters were tested and showed that they improve the search precision [12]; however, both have been modified to conform to this study's needs. Namely, the subject heading for randomized control studies (RCTs) was removed from original filters, since we did not want to limit the retrieved studies only to RCTs. Limits that are part of NHS EED, MEDLINE G, and EMBASE G filters included humans and publication type. The final search was not limited by language.

Since the early economic evaluation concept is not well defined and it is not included in the MeSH controlled vocabulary, an initial scoping search was performed in order to identify potential candidate keyword terms as well as MeSH subject headings. A set of seed articles was used to develop the MeSH terms and keywords used in the literature search [2,5-8,13-22]. These articles were selected because they are commonly cited and highly relevant to early health technology assessment or early economic evaluation. The relevant MeSH terms harvested from the seed articles include ‘device approval,’ ‘diffusion of innovation,’ and ‘decision support techniques.’

From the seed articles, we extracted the cited keywords and other keywords that are deemed relevant to the aspects of early economic evaluation. Table 1 describes these aspects and the associated keywords. The first aspect is that the evaluation is conducted at an early phase in the development of the emerging technology. The objective of an early evaluation is to examine the likely cost-effectiveness of the technology. The evaluation is to provide decision support for product and policy development. There is limited supporting evidence regarding the effectiveness of the technology. Lastly, early evaluation may have an influence on the design and future performance of the technology.

**Table 1:** Aspects of early economic evaluation and associated keywords used in the literature search

**Aspects of early economic evaluation**	**Keywords**
The objective is to examine the (likely) cost-effectiveness of the emerging technology	(Like$ OR potential).tw. adj2 (effect$ OR safety OR cost-effect$ OR efficiency OR impact).tw.
	headroom.tw. OR (effect$ adj2 gap).tw.
	(earl$ OR iterative$ OR continu$ OR ongoing OR ‘on-going’ OR dynamic$ OR ex$ante OR pre$develop$ OR constructive).tw. adj3 (econom$ OR ‘health econom$’ OR pharmacoeconom$ OR analys$ OR evaluation OR frame$ OR approach$ OR assessment$ OR Bayesian OR ‘health technology assessment’ OR ‘technology assessment’).tw.
	‘development-accompanying’.tw.
The evaluation is to provide decision support for different stakeholders involved in the development and diffusion of the emerging technology	(‘early$stage’ OR earl$).tw. adj2 decision?.tw.
	(priority adj2 set?).tw. OR decision.tw adj2 (gate$ OR point$).tw.
	(product$ adj2 development$).tw. OR (polic$ adj2 development$).tw.
	(‘in-licens$’ OR ‘in licens$’ OR inlicens$).tw.
	‘only in research’.tw. OR ‘only with research’.tw. OR ‘in the context of research’.tw. OR (cover$ adj2 evidence).tw.
	((interim OR condition$ OR ((dependent OR dependant OR contingent) adj (upon OR on)) OR restrict$) adj2 (fund$ OR financed OR reimburse$ OR cover$ OR approv$ OR list$ OR access$ OR licens$ OR licenc$)).tw. OR ‘condition$ of coverage$’.tw. OR ‘condition$ of reimburse$’.tw. OR (pricing or commercialization).tw.
	((investment? or marketing or promot$ or progress$ or advancement) adj2 (decision$ or incentive$ or inducement$ or uncertaint$ or incertitude$ or economic$)).tw.
	‘go no-go’.tw. OR ‘go no go’.tw. OR ‘go\no-go’.tw. OR ‘go\no go’.tw. OR ‘go \ no-go’.tw. OR ‘go \ no go’.tw.
Limited supporting evidence for efficacy or effectiveness of the emerging technology	((earl$ OR scarc$ OR limit$ OR gap$ OR spars$ OR circumscrib$) adj2 (data OR evidence)).tw.
	Trial adj2 (evidence OR data) adj3 (unavailable OR available OR exist)
	((‘phase II’ or ‘phase I’ or ‘phase 1’ or ‘phase 2’) adj2 trial$).tw.(phase adj (‘1’ or ‘I’ or ‘II’ or ‘2’ or ‘IIa’ or ‘2a’)).tw.
	(evidence$) adj2 development$).tw.
Early economic evaluation has an influence on the design and future performance of the emerging technology	(‘research and development’ OR ‘R&D’).tw.
	((earl$ or mid$ or develop$ or formative$ or determinative$ or decisiv$ or design$ or concept$ or investigation$) adj2 (phase? or process$ or stage? or ‘life cycle’ or ‘lifecycle’ or cycle?)).tw.
Evaluation is conducted at a very early or early stage in the development of the emerging technology	((emerging OR earl$ OR pilot OR pre$develop$ OR new$ OR novel OR nascent OR original OR groundbreaking OR ‘ground-breaking’ OR pioneer$ OR ‘cutting-edge’ OR ‘cutting edge’ OR ‘leading-edge’ OR ‘leading edge’ OR radical$ OR trailblaz$ OR trendset$ OR seminal$ OR innovati$ or unconvention$ OR innovate$ OR emergent OR constructive OR promising OR developing OR ‘recently introduced’) adj3 (technolog$ OR drug? OR treatment? OR intervention? OR pharmaceutical? OR device? OR diagnos$ OR screen$ OR therapeutic$ OR implement$ OR development? OR service)).tw.

The search strategy has been developed by an information analyst with the review team and will be peer-reviewed by another information analyst from a systematic review research group [23]. Peer-reviewing of the literature search strategy will be conducted according to the Peer Review of Electronic Search Strategies (PRESS) statement [24]. The protocol for this systematic review is registered at PROSPERO, the International prospective register of systematic reviews (registration number CRD42013004636) [25].

Additional citations will be identified via scanning the reference lists, author searching of potentially relevant studies, and forward citation searching in Scopus. We suspect that many early economic evaluation studies are unpublished, especially those conducted by industry for internal use only. There appears to be no straightforward way to identify authors of these unpublished studies. We will therefore consider a chain-referral sampling approach to sample ‘hidden populations’ in our attempt to contact these authors [26].

In the chain-referral sampling approach, a hidden population is one in which a sampling frame (i.e., a list of all the members of the population) cannot be constructed in advance. As an alternative, new members are selected from the social network of existing members of the sample. In this approach, first a number of seeds are selected [26]. These seeds are members of the hidden population that have been identified: authors of published early economic evaluation studies who work for industry, and similar individuals identified from our contact files. The seeds are contacted and form the first stage (stage 0) of the sampling process. The seeds identify other members of the population. The members identified in the next stage (stage 1) are approached and then asked to identify other members. This process is continued until no new members could be identified.

We fully recognize that the above sampling scheme may not yield a comprehensive collection of unpublished study reports. The key barrier to access to internal reports of early evaluation may be the reluctance to share proprietary information. Possible authors will be contacted by email. We will follow up by phone with authors who contributed to unpublished studies in order to discuss their involvement and, if possible, request information about the unpublished material. For this approach, non-responders are defined as a lack of response after three attempts to contact a member of the sampling population.

### Study selection

Due to the broad inclusion criteria, screening of potentially relevant citations will be conducted by pairs of reviewers who are formally trained and experienced in economic evaluation. Full-text reports of citations that are deemed relevant by one screener will be obtained. Reasons for exclusion will be captured for studies that are deemed to be not relevant for the review. Full-text review will be conducted in pairs; disagreement will be discussed and, if necessary, adjudicated by another health economist.

### Data abstraction

Table 2 describes data elements that are going to be extracted from the included studies. First, understanding the decision context is necessary to discern the contribution of early evaluation to decision-making in product and policy development. Data pertaining to the decision context will be abstracted (e.g., who initiates the evaluation, who commissions it, the funding sources, why an evaluation is needed right now, who conducts the evaluation, and the primary target audience). Other abstracted data include the decision problem, disease classification, and target population, technology type (e.g., pharmaceuticals, medical devices, biomarkers), and technology stage (e.g., pre-clinical, phase I or II studies). Data pertaining to the methodological aspects of an early evaluation will be abstracted, especially how authors of included studies handled uncertainty in the early evaluation (e.g., probabilistic sensitivity analysis, value of information analysis).

**Table 2:** Abstracted data from included studies

	**Values**	**Source**
Decision context		
Who initiate the evaluation?	For example, policy decision makers, industry^b^, third-party payers, hospital managers/administrators, clinicians, patients or patient advocacy groups, not reported	[27]
Who commissioned it?	For example, policy decision makers, industry^b^, third-party payers, hospital managers/administrators, clinicians, patients or patient advocacy groups, not reported	[27]
What are the funding sources of the study?	For example, public, private, both, others, not reported	[27]
Why is an evaluation needed right now?	Informing product development, informing policies, not reported	[27]
Who conducted the study?	For example, academia, public, private, multiple sources, others, not reported	[27]
Primary target audience according to the decision context	For example, policy decision makers, industry^b^, third-party payers, hospital managers/administrators, clinicians, patients or patient advocacy groups, not reported	[27]
Defining the decision problem		
Decision problem	Open coding. Decision problems will be classified. For example, the primary reason for initiating the early evaluation could be product development, health policy	[28,29]
Disease or clinical problem	ICD-10 major categories (see listing in footnotes)	[30]
Target population	For example, patients, at-risk individuals, general public, unclear	[29]
Health technology		
Technology type	For example, pharmaceuticals, medical devices, diagnostic devices, other therapeutic technologies	[28]
Technology stage	For example, basic, translational research, clinical research^c^	[8]
Methodological aspects of the EE		
Perspective	For example, societal, publicly funded health-care system, third-party payers (e.g., insurance companies), not reported	[28,29]
Type of evaluations	For example, CEA, CUA, CMA, CBA, others	[28,29]
Basis of the evaluation	For example, trial-based, model-based, prospective or retrospective analysis, others	[29]
Time horizon	Numerical number, not reported	[28,29]
Adjusted for differential timing?	Numerical percentage, not reported	[28,29]
Likely effect of the new technology		
Quantification of the likely effect^a^	Open coding to identify data sources, methods, assumptions^d^	[28,29]
Valuing health outcomes		
Valuation of the likely effect^a^	Open coding to identify data sources, methods, assumptions^d^	[28,29]
Resources used and costs		
Quantification of the likely impact on resources^a^	Open coding to identify data sources, methods, assumptions^d^	[28,29]
Modeling, if appropriate		
Model type	For example, decision tree, state transition (e.g., Markov), discrete event simulation, dynamic transition model, others	[28,29]
Handling of variability and uncertainty		
Scenario analysis (e.g., structural uncertainty)	Yes, no	[28,29]
Sensitivity analysis (e.g., one-way, multiple-way)	Yes, no	[28,29]
Probabilistic sensitivity analysis	Yes, no	[28,29]
Value-of-information analysis	Yes, no	[28,29]
Presenting results of the economic evaluation		
According to the authors, are results likely to be influential to the decision problem?	Yes, no, unclear	[28,29]
According to the reviewers, are results likely to be influential to the decision problem?	Yes, no, unclear	[28,29]

^a^Definition of treatment effect of a technology can be summarized as the difference between the duration and state of health or HRQL (including the impact of any adverse effects of treatment) that would be experienced on average by patients receiving the technology and that experienced by the same group were they to receive alternative care; ^b^industry would include pharmaceutical, device, or genomic manufacturers; investors or inventors;^c^technologies are ready for diffusion after regulatory approval, certification of laboratory quality, or marketing authorization; ^d^text coding and review of text descriptions across included studies to derive a broad classification for data sources (e.g., expert opinion, individual patient data, administrative databases, clinical registries), methods used (e.g., systematic review, meta-analysis, RCT, non-RCT) and types of assumptions (e.g., based upon basic science, early clinical experience). *CEA* cost-effectiveness analysis, *CUA* cost-utility analysis, *CMA* cost-minimization analysis, *CBA* cost-benefit analysis, *QALY* quality-adjusted life years, *HRQOL* health-related quality of life, *ICER* incremental cost-effectiveness ratio, *CE* cost-effectiveness, *PSA* probabilistic sensitivity analysis.

Open text coding will be used to capture the following descriptions: the likely effectiveness of a new technology and how the associated health consequences and resources used were measured and valued. These descriptions will be used to discern the data sources, methods, and assumptions involved in the early evaluation. We will assess as to whether the results of an included study are adequate to answer the main study questions or objective, according to authors of included studies and according to the reviewers. A pair of reviewers using the Dedoose software for qualitative literature review will abstract each included study independently [31].

### Evidence synthesis

Characteristics of early economic evaluation studies will be summarized according to the various aspects that define early evaluation (Table 2). Given the qualitative nature of this systematic review, there will be no quantitative evidence synthesis. Unpublished studies obtained from the chain-referral sampling will be summarized separately because the representativeness of this sample of unpublished studies (relative to the population of early economic evaluation conducted to support internal decisions) is uncertain.

## Discussion

This protocol describes a systematic review of published and unpublished studies of early economic evaluation of regulated health technologies, when the supporting evidence for their effectiveness is not fully established. The aim is to understand current practice regarding the use of early economic evaluation to inform decisions in the development of emerging technologies, including decisions regarding the allocation of private and public resources for the development of these technologies and decisions regarding the planning and design of research programs to collect additional evidence to support regulatory and reimbursement requirements. This systematic review will summarize the methods used in early economic evaluation and provide data regarding the contribution of early economic evaluation to decision-making for product and policy development.

Our systematic review is timely. Conceptual discussion of early HTA over a decade ago has evolved into concrete implementations over recent years [6,21,32-34]. As we understand it, early HTA initiatives now exist in the UK, the Netherlands, Austria, and Ontario [35,21,37]. These initiatives facilitate the collaboration between private enterprises, public sectors, and academia in the development of selective technologies that address the needs of patients and health systems and, to varied extent, investment expectations [35]. Early economic evidence may facilitate decisions regarding the selection of promising technologies and the complex trade-offs between public and private investments [36].

Economic evaluation is essential for health technology assessment. Ijzerman and colleagues review previous work and methods of early health technology assessment and propose a conceptual framework for early assessment that acknowledges the different phases in technology development and the specific decision problems faced by different decision makers [8]. They review a range of tools to identify and evaluate technologies in development, most notably early health economic modeling. They suggest that early HTA is an emerging field of research that will likely become more important because of the increasingly complex trade-offs between investments and access in medical product development.

The method outlined in this protocol is sometimes referred to as a meta-epidemiological investigation. In particular, there are well-established precedence regarding the use of systematic review to investigate factors affecting the conduct and reporting of trials, systematic reviews, and economic evaluations of health interventions [38-45]. By adapting a systematic approach to our investigation, our aim is to collect relevant data from a representative sample of all early economic evaluation studies and, therefore, reliably discern the contribution of early economic evaluation to product and policy development.

## Competing interests

The authors declare they have no competing interests in relation to the planned systematic review.

## Authors’ contributions

BP participated in conception and design of the project, piloting the protocol with a literature search from January 2010 to March 2013, preparing the results of the pilot review for a presentation at the panel discussion on early Health Technology Assessment at the Canadian Drugs and Technologies in Health Symposium 2013 in St John’s Newfoundland and Labrador Canada, drafting and revising the manuscript. HATT participated in designing the project, piloting the protocol, preparing the presentation, drafting and revising the manuscript. DH participated in designing the project, piloting the protocol, preparing the presentation, drafting and revising the manuscript. PP participated in conception and design of the project, piloting the protocol, preparing the presentation, drafting and revising the manuscript. JB participated in designing the project, piloting the protocol, obtaining peer-review of the literature search, conducting the literature search, registering the protocol, and drafting and revising the manuscript. FM participated in conception and design of the project and revising the manuscript. VR participated in conception and design of the project and revising the manuscript. WC participated in conception and design of the project and revising the manuscript. ACT participated in conception and design of the project and revising the manuscript. MP participated in conception and design of the project and revising the manuscript. MK participated in conception and design of the project, revising the manuscript and being responsible for the direction and quality of the project. All authors approved of the final manuscript.
